# Mapping vulnerability for increased COVID-19 susceptibility and worse outcomes: a scoping review

**DOI:** 10.3389/fpubh.2024.1432370

**Published:** 2024-10-10

**Authors:** Rodrigo de Macedo Couto, Daniel Gonçalves dos Santos, Patrícia Rodrigues Sanine, Andrea Pires dos Santos, Louise Bach Kmetiuk, Alexander Welker Biondo, Alexandra Crispim Boing

**Affiliations:** ^1^Department of Public Health, Federal University of Santa Catarina, Florianópolis, SC, Brazil; ^2^Faculty of Public Health, University of São Paulo, São Paulo, SP, Brazil; ^3^Graduate Program of Public Health, State University of São Paulo, Botucatu, SP, Brazil; ^4^Department of Comparative Pathobiology, Purdue University, West Lafayette, IN, United States; ^5^Zoonosis Surveillance Unit, City Secretary of Health, Curitiba, PR, Brazil; ^6^Department of Veterinary Medicine, Federal University of Paraná (UFPR), Curitiba, PR, Brazil

**Keywords:** COVID-19, pandemics, public health policies, SARS-CoV-2, vulnerability

## Abstract

**Introduction:**

Infectious diseases can spread rapidly in the globalized world, and the complex intersection of individual, social, economic, and cultural factors make it difficult to identify vulnerabilities in the face of pandemics.

**Methods:**

Therefore, this study aimed to identify vulnerability factors to infection and worse outcomes associated with COVID-19. This is a scoping review study of six databases that selected publications between 2019 and 2023, focusing on individual, social, and programmatic dimensions of vulnerability. The results were recorded in a spreadsheet and analyzed, considering the interrelationships among these dimensions.

**Results:**

A total of 45 articles were included in the review. Content analysis was conducted using the theoretical framework of health vulnerability, which divides vulnerability into individual, social, and programmatic dimensions. Race/ethnicity, homelessness, incarceration, socioeconomic level, food insecurity, and remote areas were classified as social dimensions. On the other hand, cancer, cardiovascular disease, HIV/AIDS, alcoholism, advanced age, obesity, mental disorders, diabetes, kidney disease, and pregnancy were classified as individual dimensions. None of the publications found explicitly mentioned programmatic vulnerabilities.

**Discussion:**

The research found that social vulnerabilities reinforce individual vulnerabilities, creating a vicious cycle. In addition, programmatic vulnerabilities reinforce this relationship. This study emphasizes that public policies should address these different dimensions of vulnerability. It suggests that this information should be incorporated into health surveillance and future decision-making to face new pandemics.

**Systematic review registration:**

https://archive.org/details/osf-registrations-wgfmj-v1.

## 1 Introduction

History records several diseases characterized as pandemics or major epidemics. However, the greater ease of movement between countries compared to other times suggests that they will occur more frequently and on a larger scale, like what it was experienced with COVID-19 (1) significant deficiencies in surveillance networks, such as a coordinated national response, timely notification, and identification of circulating strains, facilitate this spread (2). COVID-19 is a highly communicable acute respiratory disease responsible for a large number of deaths. Although full recovery is expected in most infected individuals, there is evidence of a wide range of persistent clinical, psychological and/or physical manifestations that can affect various body systems in the medium and long term (1).

The concept of vulnerability in the context of the COVID-19 pandemic is multifaceted. Vulnerable groups are those disproportionately exposed to risk, but this categorization may change over time, especially in response to policy measures. While it is generally recognized that older people and individuals with underlying health conditions are at increased risk, the definition of vulnerable expands to encompass a spectrum of socioeconomic groups. For many, recommended preventative measures such as social distancing and hand washing are challenging to implement because they live in densely populated areas with inadequate housing, sanitation, and access to drinking water. Besides, these individuals may also face malnutrition, non-communicable diseases, and infectious diseases (3).

While the literature has provided critical insights into health inequalities during the pandemic, there is a notable gap regarding studies exploring the complex intersection of individual, social, economic, and cultural factors contributing to vulnerability. Identifying the constraints that promote these adverse outcomes, which are associated with an increased risk of clinical worsening and death, is paramount.

Thus, based on the assumption of multidetermination—which recognizes the influence of vulnerabilities (individual and social) in the illness process (4) it is believed that the identification of different situations of vulnerability to COVID-19 can favor the planning of strategies that meets the specific needs of each of them, especially for the definition of population groups that should be prioritized in prevention and assistance actions for COVID-19 (5).

From the perspective of overcoming challenges in planning health practices, the objective was to assess how well the literature addresses individual, social, and programmatic dimensions, identifying issues that characterize population groups most vulnerable to SARS-CoV-2 infection or poor disease outcomes.

## 2 Materials and methods

This scoping review study rigorously applied the principles of the scientific method to systematize available literature, adhering to the transparency and reproducibility criteria recommended by PRISMA-ScR (6). All the rigor of systematizing the principles of the scientific method and evidence synthesis was maintained, including the transparency and reproducibility criteria (7) recommended by PRISMA-ScR (8). The present study was registered in the Open Science Framework (OSF) (9), a scoping review platform allowing open collaboration in research, review protocols, data sharing, and project organization. No registration was made in the Prospero database, a platform for registering systematic reviews and not for scoping reviews as herein.

To address this central issue, the following research question was adopted: What characterizes population groups most vulnerable to getting infected with COVID-19 or to poor disease outcomes as recorded in the scientific literature? The acronym PICO (Population, Intervention, Comparison and Outcome) has several variants, one of which was designed for scoping reviews, applied as PCC (Population/Problem, Concept, Context) (10). It was built according to the acronym P (population/problem)—people with COVID-19; C (concept)—vulnerability to illness and poor disease outcomes; C (context)—scientific evidence.

To collect these pieces of evidence, searches were carried out on the main databases and data repositories, including PubMed/Medline, Embase, Scopus, Web of Science, Lilacs, and Scielo. With the support from a librarian experienced in review studies, a search strategy was developed to use the MeSH (Medical Subject Headings) search terms on the PubMed database and adapted it for other databases, according to the languages used in the periodicals (Supplementary Table 1).

The searches considered the fields title, abstract, or keyword and were carried out in December 2023. Due to the authors' language proficiency, only Portuguese, English, Spanish, Italian, and French publications were included. The study focused on publications during the pandemic; original studies and/or reviews published between 2019 and 2023. Both qualitative and quantitative study designs were accepted. We considered the following as poor outcomes: symptomatic illness, hospitalization, need for intensive care, injuries, and death. Publications such as comments, editorials, case reports, or summaries of scientific events were not included. Publications that addressed specific characteristics of the pandemic impact on cities or people's way of life were also excluded, as well as prevention and coping strategies for the disease, communication tools, political discussions, and exclusively clinical studies aimed at diagnosis or treatment.

After eliminating duplicates, the inclusion and exclusion criteria were calibrated between the three reviewers (extraction: RMC and DGS; validation: PRS). The reviewers carried out the selection by title and abstract, followed by the full-text reading of those eligible studies. At the end of this process, the matrices containing the study collection were exchanged between the extractors so that both could check the result. Disagreements were analyzed by the validation reviewer and decided by the three reviewers when reaching a consensus. The reference management and citation formatting were done using Endnote^®^ software and exported for processing on Rayyan QCRI^®^.

The extracted content was subjected to the same checking and validation procedure and duly recorded in an electronic spreadsheet shared among the reviewers. The spreadsheet was built on Excel^®^ program containing the following fields: ID, type of study, title of publication, year of publication, name of the journal, authors, URL, language, study population, characteristics (individual, collective, or contextual) that presented themselves as vulnerabilities to getting sick with COVID-19.

The content analysis was carried out in light of the theoretical framework of vulnerability in health, which separates it into three dimensions—individual, social, and programmatic (4). For the author, the individual dimension corresponds to individual behaviors and people's way of life; the social dimension refers to the conditions in which people live, such as access to goods, services, education, and culture, including racial, gender, and religious issues, among others. The programmatic dimension encompasses effective and democratic access to institutionally guaranteed resources, such as health, education, and social assistance policies.

However, considering that in addition to the existing overlap between these dimensions, in the real context, they are interrelated, articulating with each other in a dialogic manner (4), these dimensions were connected according to the similarity of the content identified in the data, which allowed identifying them as factors of vulnerability to illness and/or unfavorable outcomes related to COVID-19.

## 3 Results and discussion

**Figure 1:**
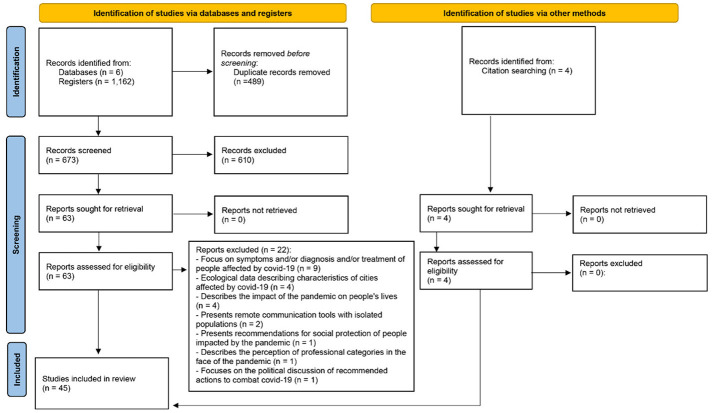
Flowchart of the selection process of publications included in the study. Source: PRISMA 2020 (8).

Database searches identified a total of 1,162 records, out of which 489 (42.1%) were removed due to duplication, remaining 673 articles. Of this remaining sample, 610 (90.6%) were not selected during the first screening (titles and abstracts). once they did not meet the inclusion criteria. Consequently, 63 articles proceeded to the eligibility stage, but 22 (34.9%) were excluded for meeting the exclusion criteria. Additionally, from reading the references of the selected articles, four more articles were identified for full-text reading, and all of them (100.0%) were selected for the study. In the end, 45 articles were included in the review. A flowchart with the records found, selected, and excluded is represented in Figure 1.

The majority of identified publications used the English language (43/45; 95.5%). Most of them were published in 2021 (18/45; 40.0%), followed by 2022 and 2020 (13/45; 28.9%, and 11/45; 24.4%, respectively), and the smallest portion was published in 2023 (3/45; 6.7%). This result was expected since there is a preference for scientific publications in the English language at the international level, and as this is a new topic, most studies were concentrated shortly after the emergence of the first cases of COVID-19 worldwide.

A series of factors signaled different vulnerabilities to illness and/or unfavorable outcomes related to COVID-19. All these factors and the respective characteristics of the publications can be seen in Table 1.

**Table 1:** Characterization of publications according to their respective first authors, year, language, vulnerability to illness, and/or unfavorable outcomes related to COVID-19, dimensions classification and country of the study.

**References**	**Year**	**Language**	**Vulnerability to illness and/or unfavorable outcomes related to COVID-19**	**Dimensions**	**Country**
Khanijahani et al. (16)	2021	English	Race/ethnicity; education level and income; immigrants	Social	USA; England; Brazil; UK; Sweden; Ireland; Kuwait; Spain
Boserup et al. (17)	2020	English	Race/ethnicity; education level and income; socioeconomic level; use of public transportation during the pandemic	Social	USA
de Oliveira et al. (18)	2020	Portuguese	Race/ethnicity	Social	Brazil
Kirksey et al. (24)	2021	English	Race/ethnicity	Social	USA
Glance et al. (19)	2021	English	Race/ethnicity; work occupation	Social	USA
Green et al. (20)	2021	English	Race/ethnicity; work occupation	Social	USA; UK; Iran; Italy
Islamoska et al. (21)	2022	English	Race/ethnicity; work occupation	Social	Denmark
Lee and Ahmed (22)	2021	English	Race/ethnicity	Social	USA
Khanijahani et al. (16)	2022	English	Race/ethnicity	Social	USA
Selden and Berdahl (15)	2020	English	Race/ethnicity; education level and income; work occupation	Social	USA
Papageorgiou et al. (23)	2020	English	Race/ethnicity; comorbidities and other preexisting health conditions	Social	UK
Nguyen et al. (25)	2022	English	Race/ethnicity	Social	USA; UK
De Hert et al. (55)	2022	English	Race/ethnicity, socioeconomic, education, inaccessibility to services	Social	USA; Brazil; Indonesia; India, Sweden
Alves et al. (27)	2021	English	Race/ethnicity including indigenous; comorbidities and other preexisting health conditions	Social	Brazil
Flores-Ramírez et al. (28)	2021	English	Race/ethnicity including indigenous; comorbidities and other preexisting health conditions	Social	Latin America and Caribbean
Salerno et al. (26)	2020	English	LGBTQIA+ Community	Social	USA
Ahillan et al. (29)	2023	English	Homeless people; development of comorbidities and other health conditions	Social	Usa, Canada, Belgium
Esposito et al. (30)	2022	English	Incarcerated populations	Social	Canada; Honduras; USA; 47 European countries; Brazil; Italy; UK; Ireland; Spain; Argentina; Chile; Colombia; Mexico
Sánchez et al. (31)	2020	Portuguese	Incarcerated populations	Social	Brazil
Arndt et al. (33)	2020	English	Education level and income; socioeconomic level; food insecurity	Social	South Africa
Bloem and Farris (32)	2020	English	Education level and income; socioeconomic level; food insecurity	Social	Sub-Saharan Africa and India
Jacobs et al. (34)	2022	English	Socioeconomic level	Social	USA
AI Meslamani et al. (36)	2021	English	People living in rural or remote areas	Social	Egypt
Joseph et al. (38)	2023	English	People living in rural or remote areas; socioeconomic	Social	India
Morante-García et al. (37)	2022	English	People living in rural or remote areas; income and access to drinking water	Social	Spain
Liu et al. (39)	2020	English	Comorbidities and other health conditions: cancer	Individual	China
Parise et al. (40)	2022	English	Comorbidities and other health conditions: cancer	Individual	USA
Kwok et al. (35)	2021	English	Comorbidities and other health conditions: cardiovascular diseases	Individual	Not appliable
Soumya et al. (43)	2021	English	Comorbidities and other health conditions: cardiovascular diseases	Individual	Not appliable
Garcia et al. (45)	2022	English	Comorbidities and other health conditions: HIV/Aids	Individual	Latin America and Caribbean
Nomah et al. (44)	2021	English	Comorbidities and other health conditions: HIV/Aids	Individual	Not appliable
Lesko and Bengtson (46)	2021	English	Comorbidities and other health conditions: HIV/Aids, alcoholism, mental disorders, structural vulnerabilities	Individual	Not appliable
Bansod et al. (48)	2021	English	Advanced age	Individual	Not appliable
Chen et al. (47)	2021	English	Advanced age	Individual	Not appliable
Martínez-Payá et al. (49)	2022	English	Advanced age, people living in nursing homes	Individual	USA; Spain; Brazil; Canada; Saudi Arabia; France; Japan
Kwok et al. (35)	2020	English	Comorbidities and other health conditions: obesity	Individual	Not appliable
Yu et al. (51)	2021	English	Comorbidities and other health conditions: obesity	Individual	USA; Italy; China; UK; Germany; France; Mexico; Israel; Brazil
Tamara and Tahapary (50)	2020	English	Comorbidities and other health conditions: obesity	Individual	China; USA; France
Bertolini et al. (52)	2023	English	Comorbidities and other health conditions: mental disorders	Individual	33 countries from America, Asia, Europe and Oceania
Tenenbaum et al. (53)	2021	English	Comorbidities and other health conditions: mental disorders	Individual	Not applicable
Murphy et al. (54)	2021	English	Comorbidities and other health conditions: mental disorders	Individual	USA; Canada; Italy; Spain; China; Switzerland; UK; India; Australia; Ireland; Germany
De Hert et al. (55)	2022	English	Comorbidities and other health conditions: mental disorders	Individual	Denmark; USA; Israel; South Korea; France; Spain; UK
Bigdelou et al. (41)	2022	English	Comorbidities and other health conditions: cancer, cardiovascular disease and diabetes	Individual	Not appliable
Chagas et al. (56)	2021	English	Comorbidities and other health conditions: kidney diseases	Individual	Not appliable
Dashraath et al. (57)	2020	English	Pregnancy	Individual	Not appliable

Source: scoping review, 2023.

Monthly household income has been used to compare acceptance of COVID-19 vaccination, with Brazil and Chile presenting the highest acceptance rates when compared to other countries (11). Besides, this study has found that country, age group, religion, comorbidities and changes in salary during the pandemic were also associated to varying degrees of vaccine acceptance, with. Middle East and North African countries such as Egypt, Tunisia and Iran presenting the overall lowest vaccination acceptance rates (11). Such vaccine hesitancy in low and middle-income countries has shown Brazil and Chile among the lowest hesitancy vaccination rates with 6.3 and 6.8%, respectively (12). Low and middle-income countries have also presented several degrees of perception risk of becoming infected and dying from COVID-19, as well as different beliefs on social distancing and face masks as preventive measures for infection (13, 14).

As the study herein was focused on social vulnerability rather than overall economic indexes such as Gross Domestic Product (GDP), the quality of life (such as poverty) and areas where people lived were considered for analyses, with a lack of specific studies comparing social vulnerability to COVID-19 susceptibility. Although vaccination acceptance and hesitancy rates should be considered as part of infection and susceptibility, previous results have shown a high overall vaccination acceptance and low hesitancy in Brazil. As such findings may not represent within-country social disparities, leading to infection and worse outcomes, the present study aimed to approach COVID-19 susceptibility through social vulnerability instead overall economic status of the country.

### 3.1 Social dimension

#### 3.1.1 Racial and ethnicity

Racial/ethnic groups were particularly vulnerable during the COVID-19 pandemic. SARS-CoV-2 transmission is strongly associated with the history and socioeconomic characteristics of these individuals, leading to a higher risk of infection and death (15–23). These groups typically have a higher prevalence of pre-existing comorbidities, such as diabetes and systemic arterial hypertension, leading to a higher likelihood of complications (23). In addition, studies have identified associated factors such as low educational attainment, which could lead to the misunderstanding of preventive measures; poor housing conditions, such as overcrowded households, which favors transmission and make it difficult to maintain social distancing; and low family income, which affects their nutrition and housing conditions (15–17).

There are also issues related to substantial disparities in access to healthcare, resulting in low hospitalization rates combined with higher mortality rates (17). In countries without universal healthcare systems, individuals often lack health insurance and reside in cities or regions with unequal access to medical resources, such as hospitals with adequate availability of intensive care beds (19). Regarding employment, these individuals often work in essential services such as healthcare and food provision with public-facing roles, increasing their exposure to virus infection without the ability to working from home (15, 19–21).

In the case of vulnerability among the Black population, there are also issues related to racial discrimination and structural racism (18, 24), which have intensified the mental health challenges imposed by the pandemic and offered barriers to access healthcare (25). For immigrant populations, language barriers also posed communication difficulties when their language differed from that adopted by the country (16). Another subgroup of these groups includes LGBTQIA+ individuals, a population already known to face social disadvantages and health disparities. Often devoid of health insurance and residing in poverty, these individuals experience exacerbating harmful effects of the pandemic (26).

Indigenous populations have a high degree of vulnerability to COVID-19. These individuals typically have a higher prevalence of other conditions such as respiratory diseases, diabetes, and hypertension, as well as high rates of malnutrition and obesity. They also face social issues related to income sources. Additionally, it should be noted that Indigenous populations have many sociocultural specificities that influence their relationship with the environment in which they live, their way of life, and the health-disease process. An example is the high number of residents per household, the sharing of personal utensils, and collective ceremonies with large gatherings of people (27).

Historically, issues related to territorial vulnerability are also highlighted. A large portion of the indigenous population suffers from the devastation of their lands due to lack of demarcation, illegal invasion, mining, and deforestation. Being located in remote areas, there is great difficulty in transferring patients who need healthcare attention in medium and high complexity systems, in addition to having few healthcare structures, lack of inputs, equipment, lack of training, and high turnover of professionals (27).

There is also a high prevalence of other infectious diseases among Indigenous populations, such as tuberculosis (TB). Due to poverty, this disease continues to disproportionately affect Indigenous peoples worldwide. Individuals infected with TB and COVID-19 may have worse treatment outcomes, especially if TB treatment is interrupted. Regarding other communicable diseases, there is a close relationship between populations and the occurrence of diseases such as HIV/AIDS. In this sense, various indigenous cultures present a context of sexuality as part of creation connected to ancestral traditions within the life cycle, increasing the risk of sexually transmitted diseases. Furthermore, other diseases such as malaria, dengue, Zika, chikungunya, and Chagas disease are especially significant in Indigenous communities living in tropical and subtropical areas (28).

#### 3.1.2 Homelessness

People living in street situations constitute a heterogeneous population group, but they share poverty, weakened family ties, and a lack of regular housing. They typically use public facilities and degraded areas as temporary or permanent living and subsistence spaces, making them more prone to come into contact with people infected with COVID-19. Moreover, in this situation, they are not able to comply with preventive recommendations (29).

These individuals commonly have other determinants such as compromised immune systems and high rates of comorbidities, presenting excess mortality from chronic cardiovascular and respiratory diseases, contributing to an increased risk of adverse outcomes from COVID-19. Increased rates of mental health conditions (e.g., depression and schizophrenia) also stand out compared to the general population, which may affect their ability to adapt to measures implemented to prevent infection spread during the pandemic (29).

#### 3.1.3 Incarceration

Another subgroup of these groups includes LGBTQIA+ individuals, a population already known to face social disadvantages and health disparities. Often devoid of health insurance and residing in poverty, these individuals experience exacerbating harmful effects of the pandemic (30).

Considering that these individuals are usually subjected to overcrowded facilities with poor hygiene and that the main preventive measures implemented during the pandemic included social distancing and handwashing practices, it is accepted that few or no measures have been effective in the context of prison health. Furthermore, the lower level of education among the incarcerated population also stands out, contributing to a poor understanding of preventive measures (31).

#### 3.1.4 People living in poverty and people suffering from food insecurity

Poverty is caused by a lack of access to income and can impact aspects of life such as health, including hunger, malnutrition, stigma, and lack of access to essential services. People living in poverty may not have housing and may have difficulty practicing physical distancing to protect themselves from infections or self-isolation to protect others. Similarly, people living in poverty and not having access to clean water are unlikely to have water for handwashing. Even where some of these behaviors are possible, mass communication methods (e.g., through television or social media) are less likely to reach these audiences, preventing access to information on staying safe (32, 33).

Individuals who earn income through the informal economy generally receive low wages, have longer daily working hours, work in hazardous conditions, and lack of social security. COVID-19 caused a sudden and significant decrease in jobs in the informal sector, exposing individuals and their families to higher levels of financial insecurity and poverty (32–34). Commonly, people in these conditions have communicable diseases such as TB and HIV/AIDS (37).

Low-income families do not have the means to buy automobiles. Interestingly, families without of private vehicles have been associated with higher mortality from COVID-19. This finding indicates that use of public transportation is a potentially important risk factor, facilitating virus transmission and constituting a source of infection for people who depend on this service (17).

Individuals with low income have little access to healthy food and usually have more children. Studies indicate that families with more children experienced higher food insecurity during the pandemic, possibly due to difficulty providing adequate food for all family members (34).

It is worth emphasizing the role of micronutrients and vitamins in COVID-19, which has drawn attention from the scientific community. Vitamins, including A, B6, B12, C, D, E, and folate, and micronutrients including zinc, iron, selenium, magnesium, copper, and omega-3 fatty acids play important functions and complementary roles in supporting the immune system. Vitamin D deficiency, in particular, has been pointed out as a potential contributor to susceptibility to COVID-19 (35). Thus, people suffering from food insecurity are even more susceptible to infection by the new coronavirus SARS-CoV-2 (32, 33).

#### 3.1.5 People living in rural and remote areas

People living in rural and remote areas are at higher risk of acquiring and spreading COVID-19 due to various factors, such as physical barriers (distance), financial barriers (transportation costs), and language barriers (minority populations such as Indigenous peoples), which prevent them from accessing healthcare early. These populations may also be less easily reached through standard communication channels (36).

Farmers and fishermen are examples of these populations, and they often work in precarious conditions with little social protection. Typically, these people face challenges related to inadequate housing and lack of water and sanitation infrastructure, such as handwashing facilities (37, 38).

### 3.2 Individual dimension

#### 3.2.1 Cancer

During the COVID-19 pandemic, cancer patients were considered a highly vulnerable group due to weakened immune systems caused by both tumor growth and chemotherapy treatment. Moreover, given the confirmed nosocomial transmission of SARS-CoV-2 among patients in healthcare settings, cancer patients were more likely to be infected by SARS-CoV-2 due to contact with virus-contaminated areas, as they need to regularly visit hospitals for treatment or monitoring (39).

Thus, cancer patients not only had a higher risk of SARSCoV-2 infection but also exhibited an increased risk of severe infections. Clinical events such as admission to intensive care units, the need for invasive ventilation, greater severity, or death were more frequently observed in people with cancer than in those without cancer. Additionally, cancer patients with COVID-19 had longer hospital stays (39).

Among cancer types, hematologic cancers, including leukemia, lymphoma, and myeloma, had the highest rates of severity and mortality compared to other cancers. This is likely attributed to the reduced immune function of white blood cells in the presence of malignant plasma cells. Lung cancer patients had the second highest rates of death, ICU admission, risk of severe or critical symptoms, and chance of invasive mechanical ventilation use. Metastatic cancer (stage IV) compared to non-metastatic cancer presented higher risks of severe conditions and/or unfavorable outcomes (40).

Colorectal cancer, prostate cancer, bladder cancer, and breast cancer also had implications of severity for COVID-19 cases, but with less intensity than hematologic and lung cancers. Studies on COVID-19 and pancreatic cancer are limited at the moment due to limited data availability in small samples and the rarity of pancreatic cancer (40).

#### 3.2.2 Cardiovascular diseases

The infection by the SARS-CoV-2 virus can directly affect the cardiovascular system, causing complications such as myocarditis, arrhythmia, cardiogenic shock, heart failure, and thromboembolic events. These events can lead to the development of cardiovascular disorders such as acute coronary syndrome and venous thromboembolism (41).

The SARS-CoV-2 virus utilizes the Angiotensin-Converting Enzyme 2 (ACE2) receptor to enter cells, and since this receptor is present in the heart and vascular endothelial cells, these organs end up being sites of viral replication (42). The binding of SARS-CoV-2 to the ACE2 receptor causes acute myocardial injury through alterations in signaling pathways. ACE2 protects the heart against the renin-angiotensin-aldosterone system (RAAS) activation by converting angiotensin II into angiotensin. Angiotensin II is a vasoconstrictor pro-inflammatory mediator that damages the capillary endothelium, while angiotensin is a vasodilator. However, virus entry causes negative regulation of ACE2 and increases angiotensin II levels, leading to increased cardiac damage (43).

Hypertension, often present in patients with cardiovascular diseases, can exacerbate SARS-CoV-2 infection. Excessive activation of monocytes, caused by vascular endothelium in hypertensive patients, leads to uncontrolled release of cytokines, resulting in inflammation and cardiac dysfunction, including fulminant myocarditis. Hypertension can also cause dysfunction of CD8+ cells, and the use of immunotherapeutic medications related to hypertension treatment can increase cardiac and systemic inflammation. Additionally, hypertension can lead to hyperinflammation of the respiratory pathways and slow viral clearance, contributing to the severity of COVID-19 infection (41).

COVID-19 also affects blood coagulation, leading to increased D-dimer levels, prolonged prothrombin time, and reduced platelet count. These coagulation abnormalities increase the risk of thromboembolic events, contributing to the severity of the disease in patients with cardiovascular diseases (41).

#### 3.2.3 HIV/AIDS

Although some studies find a higher mortality rate from COVID-19 in people living with HIV (PLHIV), it is believed that this finding is not due to the infection itself but rather to the correlated burden of other comorbidities and life conditions (44, 45). The prevalence of some comorbidities, such as alcohol, tobacco, and drug use, is higher among PLHIV. Additionally, PLHIV take various medications, leading to associated comorbidities such as cardiovascular and renal diseases. It is also worth considering that HIV disproportionately affects marginalized groups such as racial or ethnic minorities, men who have sex with men, transgender individuals, and people with a history of incarceration (44, 46).

Another factor affecting PLHIV and elevating their vulnerability is that this population requires continuous healthcare services, exposing them more to infected individuals. Moreover, with the interruption of many services, there has been difficulty accessing treatment and monitoring cases and the cessation of preventive campaigns (44, 45).

#### 3.2.4 Alcoholism

Most of the time, alcohol consumption is considered harmful to health, with effects that include interference with the communication pathways of the nervous system, cardiovascular conditions such as cardiomyopathy and arrhythmia, and weakening of the immune system. Those who chronically consume more than 20–40 g/day of alcohol (reference for women) or 30–60 g/day (reference for men) are at higher risk of COVID-19 infection (41).

Alcohol intake increases the risk of pneumonia due to increased permeability of the alveolar barrier, thereby increasing the likelihood of viral infection. Additionally, there are effects of alcohol on the immune system, also increasing the risk of malnutrition and, over time, advanced alcohol-related liver diseases. It is worth emphasizing that chronic alcohol consumption diminishes the body's antibody response after vaccination (41).

#### 3.2.5 Advanced age

Aging is considered a significant risk factor for death from COVID-19, being related to the dysregulation of immune function, known as immunosenescence. Additionally, the reason for this disproportion in infection and severity in older individuals can also be attributed to the fact that they suffer from many chronic diseases and disabilities, some of them severe in nature (47, 48).

Another aspect that should be considered, contributing to the high infection rate in the older adult, is the fact that many older adult individuals live in nursing homes, which are among the hardest-hit locations in terms of high transmissibility and mortality. This increased vulnerability is related to the characteristics of the nursing homes themselves that facilitate infection spread (closed systems, proximity between residents and staff). Close interaction among individuals in nursing homes increases the risk of death in institutionalized older adult compared to individuals with the same age-related vulnerabilities but living in more isolated environments, such as private homes (49).

#### 3.2.6 Obesity

Obesity is considered the most common vulnerability condition for COVID-19 patients under 64 years old (50). Strong scientific evidence indicates that obesity is a risk factor for more severe outcomes of SARS-CoV-2 infection. Obesity affects respiratory function through various mechanisms, such as pulmonary restriction, ventilation-perfusion mismatch, and respiratory muscle fatigue, which can lead to reduced ventilatory capacity and increased respiratory workload, reducing respiratory drive (35, 50).

Moreover, obesity is a known risk factor for thrombotic disorders, such as venous thromboembolism, including deep vein thrombosis and pulmonary embolism, cardiovascular disease, and stroke. COVID-19, in turn, is associated with significantly higher levels of D-dimer, prolonged prothrombin time and activated partial thromboplastin time, and when associated with pre-existing obesity, reflects in more severe outcomes (35).

Obesity is also associated with increased production of inflammatory cytokines such as TNF-α, interleukins, and interferons that characterize low-grade chronic inflammation and impair immune responses, both innate and adaptive. A hyperinflammatory response with high levels of interleukins and TNF-α has been associated with increased COVID-19 mortality. It is speculated that chronic inflammation in obese patients contributes to the increased mortality observed due to a potential increase in the inflammatory process in response to SARS-CoV-2 infection (35).

Obesity still poses challenges for patient diagnosis and treatment, such as low-quality diagnostic imaging, difficulties in airway management, and lack of response to prone positioning (51). Finally, it is worth noting that the presence of ACE-2 in adipose tissue means that obese individuals have more of these receptors, facilitating SARS-CoV-2 infection (the ACE-2 receptor has been recognized as a receptor for virus entry into the human body) (50).

#### 3.2.7 Mental disorders

The COVID-19 pandemic has severely affected people with pre-existing mental health disorders which have been associated with a higher likelihood of contracting SARS-CoV-2 infection and experiencing a more severe course with increased chances of death. Likely factors involved include difficulties in understanding the need to adhere to behavioral means of social distancing, associated cardiovascular comorbidities, difficulties in accessing medium and high complexity medical care, and living in shared homes, nursing homes, therapeutic communities, or being hospitalized patients, facilitating virus contact and transmission (52).

Another critical factor is the late detection of important symptoms related to mental health, as this population may often have difficulty expressing their feelings (53). Additionally, COVID-19 restrictions have compromised regular daily routines and social rhythm, thus increasing stress levels, further accelerating cortisol levels, leading to exacerbation of generalized anxiety disorder, depressive symptoms, and chronic insomnia (54).

Another interesting point for discussion in this group is the use of psychotropic medications, as the use of these medications may be an important factor associated with more severe COVID-19 cases. Some antipsychotic medications such as clozapine and valproate appear to increase susceptibility to pneumonia and pneumonia-related illnesses. Additionally, clozapine, in particular, can suppress immune function (55).

#### 3.2.8 Diabetes

Diabetes occurs in two main types: type 1, in which there is no insulin production, and type 2, in which despite insulin production, there is an inefficient response. Hyperglycemia, in combination with other risk factors, can modify immune and inflammatory processes, predisposing individuals to severe and potentially fatal COVID-19. Hyperglycemia weakens the host's defense system, compromising the function of granulocytes and macrophages and leading to lymphopenia. Mortality from COVID-19 is further increased by related diabetic complications, such as hypertension, heart failure, obesity, and chronic kidney disease (41).

#### 3.2.9 Kidney diseases

Based on the evidence currently available, COVID-19 appears to affect the kidney through different mechanisms, including direct cytopathic effects, immunological mechanisms such as immune complex deposition, indirect effects on renal tissue from other mediators, and dysfunction or injury of other organs such as the heart and lungs. These mechanisms seem to contribute to renal dysfunction and the incidence of acute kidney injury, being associated with disease severity and in-hospital mortality. Notably, ACE2 is expressed in urinary organs almost 100 times more than in respiratory organs and is used by SARS-CoV-2 as a receptor to invade host cells (56).

Individuals with chronic kidney disease, kidney transplant recipients, and patients on hemodialysis who develop COVID-19 present with greater disease severity and mortality. This population commonly has advanced age, a high prevalence of comorbidities, and immune dysfunction (56).

#### 3.2.10 Pregnancy

Mechanical changes during pregnancy increase susceptibility to infections in general, particularly when the cardiopulmonary system is affected. As pregnancy progresses, a woman's uterus expands, exerting additional pressure on the diaphragm and lungs. This change can reduce lung capacity and make it more difficult for pregnant women to breathe, which can exacerbate respiratory symptoms if they contract COVID-19 (57).

Additionally, physiological changes can also make women more susceptible to certain infections, especially changes in the immune system. Th1 and Th2 responses are two types of immune responses that are related to the activation of different types of helper T cells (CD4+ T cells) in the immune system. These cells play a crucial role in coordinating and regulating the body's immune response against pathogens such as viruses, bacteria, and other infectious agents. During pregnancy, changes occur in a woman's immune system to protect the developing fetus and prevent an excessive immune response that could be harmful to the baby. These changes can affect the balance between Th1 and Th2 responses, making pregnant women more susceptible to some infections, including viral infections like COVID-19 (57).

#### 3.2.11 Programmatic dimension

It is worth noting that none of the publications included in this study explicitly mentioned programmatic vulnerabilities. Nevertheless, it is important to consider that both individual and social vulnerabilities can be minimized by institutional actions incorporated from solid political commitments, consolidated, for example, in fiscal and financial incentives to encourage education, availability, and access to quality information to guarantee, respect and promotion of human rights, community participation in the management and planning, and supervision and evaluation of services, among others (4).

It is important to highlight that social vulnerabilities were also reported as factors that reinforced individual vulnerabilities because of the increase of pre-existing social inequities, becoming a determinant of premature mortality, independently of other clinical factors, feeding back into a “vicious cycle” of increasing vulnerabilities (58). The association between this individual and social vulnerability signals a vulnerability often fuelled by programmatic proposals. This induction may be based on the absence of actions—on the part of the State—capable of interrupting this reproduction that establishes these vulnerabilities in Society (59).

In this sense, one of the publications identified is emphatic in pointing out the need to implement social protection mechanisms to reduce health inequities. Among the recommended actions, it mentions the importance of guaranteeing access to health, education, housing, and other essential services, such as healthy food, and the need for greater engagement between these different sectors (60).

The government can take different actions and provide several opportunities to avoid programmatic vulnerabilities, including the immediate health service structuring by hiring temporary professionals to deal with the overload patient burden caused by the pandemic. In addition, the vaccine timely purchase and proper distribution may be essential to mitigate the disease spreading impacts (61). At this point, government should invest in actions to prevent vaccine hesitancy, leading to high acceptance vaccination rates. Effective strategies have included public awareness campaigns, which may clarify the vaccine benefits, demystifying its use and combating fake news. The use of social media, public announcements and partnership of community organizations can help to convey clear messages on vaccine advantages including safety and efficacy (12).

In this area of communication, clarifying the risks of coronavirus infection and reinforcing the importance of preventive measures, such as social distancing and the use of masks, have been crucial to reduce the virus transmission and protect public health (14). Campaigns by the organized community may help to address gaps in government responses and encourage higher social participation in public health measures and implementation. Community engagement strategies, such as informational meetings and education programs, may help to increase the acceptance of health guidelines and strength collaboration between governments and their citizens (61).

## 4 Limitations

Our results should be interpreted with caution since they present common limitations to scoping review studies, such as the lack of a careful assessment of the quality of publications and shortcomings regarding search locations. Furthermore, another limitation that should be considered is the specificities of each location or country, such as ethnic demographics, immigration patterns, racism, etc.

However, despite the limitations presented, it is believed that looking at these results according to the different dimensions of vulnerability (individual, social, and programmatic) allowed elucidating issues inherent to planning future interventions. Moreover, the analysis from this perspective helped highlight the extent to which the individuals have the power to protect themselves from or be exposed to certain situations of vulnerability to COVID-19 or whether more incisive interventions by the State are appropriate to act on some aspects of society (4). This study should be incorporated into decision-making and the development of public policies that address the need to prioritize some population groups in response to future pandemics.

## 5 Conclusions

The publications analyzed revealed different vulnerabilities for getting sick with COVID-19 or clinical worsening, making it possible to synthesize them into population groups that should be prioritized in similar situations. Among the social vulnerabilities, issues related to race/ethnicity, socioeconomic level, education, and housing conditions stood out. On the other hand, among individual vulnerability factors, comorbidities and other preexisting health conditions were the most frequently addressed ones, followed by age. Although none of the publications made programmatic vulnerabilities explicit, they were revealed in the absence of public policies that recognize the promotion of social equity actions as an attribute that guarantees comprehensive health care.

This study also highlighted the interrelationship between social and individual factors that is increased by programmatic vulnerabilities. Therefore, it is concluded that in the face of this unprecedented challenge, strategies for tackling programmatic vulnerabilities should be emphasized, seeking to reinforce social protection actions and combat discriminatory situations that promote social exclusion and stigmatization of people guaranteed by the State through public policies. Otherwise, such vulnerabilities will continue to overlap these population groups, feeding back into a vicious cycle of vulnerability of the subjects in a dialogic manner, which blames them by transferring responsibility for the illness to the infected person.

In summary, the scientific contribution herein may rely in the integrated analysis of the three dimensions (individual, social, and programmatic), which may have been little explored to date, particularly correlating the individual and social aspects in the programmatic dimension. This multifaceted approach may be essential for understanding vulnerability to COVID-19 in a broader context, considering both individual and societal characteristics, as well as the policies and programs that influence such outcomes. In such a scenario, this integration may be an innovative aspect improving the academic and practical discussion on COVID-19 vulnerabilities.

## Data Availability

The original contributions presented in the study are included in the article/Supplementary material, further inquiries can be directed to the corresponding author.

## References

[1] Lai CC Shih TP Ko WC Tang HJ Hsueh PR. Severe acute respiratory syndrome coronavirus 2 (SARS-CoV-2) and coronavirus disease-2019 (COVID-19): the epidemic and the challenges. Int J Antimicrob Agents. (2020) 55:105924. 10.1016/j.ijantimicag.2020.105924 32081636 PMC7127800

[2] Archer BN Abdelmalik P Cognat S Grand PE Mott JA Pavlin BI . Defining collaborative surveillance to improve decision making for public health emergencies and beyond. Lancet. (2023) 401:1831–4. 10.1016/S0140-6736(23)01009-7 37230104 PMC10202415

[3] The Lancet null. Redefining vulnerability in the era of COVID-19. Lancet. (2020) 395:1089. 10.1016/S0140-6736(20)30757-1 32247378 PMC7270489

[4] Ayres JRdCM Calazans GJ Saletti Filho HC Franca Junior I. Risco, vulnerabilidade e práticas de prevenção e promoção da saúde. Tratado de saúde coletiva (2009). Available at: https://repositorio.usp.br/item/001851472 (accessed May 12, 2024).

[5] She J Hou D Chen C Bi J Song Y. Challenges of vaccination and herd immunity in COVID-19 and management strategies. Clin Respir J. (2022) 16:708–16. 10.1111/crj.13543 36172975 PMC9539035

[6] Peters MDJ Godfrey CM McInerney P Soares CB Khalil H Parker D. The Joanna Briggs Institute Reviewers' Manual 2015: Methodology for JBI Scoping Reviews. (2015). Available at: https://repositorio.usp.br/item/002775594 (accessed May 12, 2024).

[7] Tricco AC Lillie E Zarin W O'Brien KK Colquhoun H Levac D . PRISMA extension for scoping reviews (PRISMA-ScR): checklist and explanation. Ann Intern Med. (2018) 169:467–73. 10.7326/M18-0850 30178033

[8] OSF (2011). Available at: https://osf.io/?utm_term=&utm_campaign=OSF+General&utm_source=adwords&utm_medium=ppc&hsa_acc=5222345373&hsa_cam=217339509&hsa_grp=60774351098&hsa_ad=295172720879&hsa_src=g&hsa_tgt=dsa-520694377169&hsa_kw=&hsa_mt=&hsa_net=adwords&hsa_ver=3&gclid=Cj0KCQiAo7KqBhDhARIsAKhZ4uhPvoM8JsGv8JqvAhkwb2F3wkvB0h_v7_im7nXfCYpmIjMiOSGAdtAaAij-EALw_wcB (accessed September 17, 2024).

[9] de Macedo Couto R dos Santos DG Sanine PR dos Santos AP Kmetiuk LB Biondo AW . Vulnerabilities and Covid-19: A Scoping Review. (2023). Available at: https://osf.io/wgfmj (accessed May 12, 2024). 10.3389/fpubh.2024.1432370 PMC11499102 39450391

[10] University of Leicester. What is PRISMA, and Why Do You Need a Protocol? Library and Learning Services (2009). Available at: https://le.ac.uk/library/research-support/systematic-reviews/prisma (accessed September 17, 2024).

[11] Rosiello DF Anwar S Yufika A Adam RY Ismaeil MI Ismail AY . Acceptance of COVID-19 vaccination at different hypothetical efficacy and safety levels in ten countries in Asia, Africa, and South America. Narra J. (2021) 1:e55. 10.52225/narra.v1i3.55 38450212 PMC10914086

[12] Harapan H Anwar S Yufika A Sharun K Gachabayov M Fahriani M . Vaccine hesitancy among communities in ten countries in Asia, Africa, and South America during the COVID-19 pandemic. Pathog Glob Health. (2022) 116:236–43. 10.1080/20477724.2021.2011580 34928187 PMC9132408

[13] Gachabayov M Sharun K Felsenreich DM Nainu F Anwar S Yufika A . Perceived risk of infection and death from COVID-19 among community members of low- and middle-income countries: a cross-sectional study. F1000Res. (2022) 11:345. 10.12688/f1000research.109575.1 36128553 PMC9468621

[14] Harapan H Yufika A Anwar S Ophinni Y Yamada C Sharun K . Beliefs on social distancing and face mask practices during the COVID-19 pandemic in low- and middle-income countries: a cross-sectional study. F1000Res. (2022) 11:206. 10.12688/f1000research.79534.136510264

[15] Selden TM Berdahl TA. COVID-19 and racial/ethnic disparities in health risk, employment, and household composition. Health Aff. (2020) 39:1624–32. 10.1377/hlthaff.2020.00897 32663045

[16] Khanijahani A Iezadi S Gholipour K Azami-Aghdash S Naghibi D. A systematic review of racial/ethnic and socioeconomic disparities in COVID-19. Int J Equity Health. (2021) 20:248. 10.1186/s12939-021-01582-4 34819081 PMC8611382

[17] Boserup B McKenney M Elkbuli A. Disproportionate impact of COVID-19 pandemic on racial and ethnic minorities. Am Surg. (2020) 86:1615–22. 10.1177/0003134820973356 33231496 PMC7691116

[18] de Oliveira RG da Cunha AP Gadelha AGDS Carpio CG de Oliveira RB Corrêa RM. Racial inequalities and death on the horizon: COVID-19 and structural racism. Cad Saude Publica. (2020) 36:e00150120. 10.1590/0102-311x00150120 32965376

[19] Glance LG Thirukumaran CP Dick AW. The unequal burden of COVID-19 deaths in counties with high proportions of black and hispanic residents. Med Care. (2021) 59:470–6. 10.1097/MLR.0000000000001522 33734195 PMC8132563

[20] Green H Fernandez R MacPhail C. The social determinants of health and health outcomes among adults during the COVID-19 pandemic: a systematic review. Public Health Nurs. (2021) 38:942–52. 10.1111/phn.12959 34403525 PMC8446962

[21] Islamoska S Petersen JH Benfield T Norredam M. Socioeconomic and demographic risk factors in COVID-19 hospitalization among immigrants and ethnic minorities. Eur J Public Health. (2022) 32:302–10. 10.1093/eurpub/ckab186 34718522 PMC8586727

[22] Lee IJ Ahmed NU. The devastating cost of racial and ethnic health inequity in the COVID-19 pandemic. J Natl Med Assoc. (2021) 113:114–7. 10.1016/j.jnma.2020.11.015 33339615

[23] Papageorgiou N Providencia R Saberwal B Sohrabi C Tyrlis A Atieh AE . Ethnicity and COVID-19 cardiovascular complications: a multi-center UK cohort. Am J Cardiovasc Dis. (2020) 10:455–62. 33224596 PMC7675148

[24] Kirksey L Tucker DL Taylor E White Solaru KT Modlin CS. Pandemic superimposed on epidemic: Covid-19 disparities in Black Americans. J Natl Med Assoc. (2021) 113:39–42. 10.1016/j.jnma.2020.07.003 32747313 PMC7395612

[25] Nguyen LH Anyane-Yeboa A Klaser K Merino J Drew DA Ma W . The mental health burden of racial and ethnic minorities during the COVID-19 pandemic. PLoS ONE. (2022) 17:e0271661. 10.1371/journal.pone.0271661 35947543 PMC9365178

[26] Salerno JP Williams ND Gattamorta KA. LGBTQ populations: psychologically vulnerable communities in the COVID-19 pandemic. Psychol Trauma. (2020) 12:S239–42. 10.1037/tra0000837 32551761 PMC8093609

[27] Alves JD Abade AS Peres WP Borges JE Santos SM Scholze AR. Impact of COVID-19 on the indigenous population of Brazil: a geo-epidemiological study. Epidemiol Infect. (2021) 149:e185. 10.1017/S0950268821001849 34338185 PMC8367875

[28] Flores-Ramírez R Berumen-Rodríguez AA Martínez-Castillo MA Alcántara-Quintana LE Díaz-Barriga F Díaz de León-Martínez L. A review of environmental risks and vulnerability factors of indigenous populations from Latin America and the Caribbean in the face of the COVID-19. Glob Public Health. (2021) 16:975–99. 10.1080/17441692.2021.1923777 33966608

[29] Ahillan T Emmerson M Swift B Golamgouse H Song K Roxas A . COVID-19 in the homeless population: a scoping review and meta-analysis examining differences in prevalence, presentation, vaccine hesitancy and government response in the first year of the pandemic. BMC Infect Dis. (2023) 23:155. 10.1186/s12879-023-08037-x 36918758 PMC10012317

[30] Esposito M Salerno M Di Nunno N Ministeri F Liberto A Sessa F. The risk of COVID-19 infection in prisons and prevention strategies: a systematic review and a new strategic protocol of prevention. Healthcare. (2022) 10:270. 10.3390/healthcare10020270 35206884 PMC8872582

[31] Sánchez A Simas L Diuana V Larouze B. COVID-19 nas prisões: um desafio impossível para a saúde pública? COVID-19 in prisons: an impossible challenge for public health? Cadernos *de Saúde Pública*. (2020) 36:1–14. 10.1590/0102-311x00083520 32402001

[32] Bloem JR Farris J. The COVID-19 pandemic and food security in low- and middle-income countries: a review. Agric Food Secur. (2022) 11:55. 10.1186/s40066-022-00391-4 36474782 PMC9716512

[33] Arndt C Davies R Gabriel S Harris L Makrelov K Robinson S . Covid-19 lockdowns, income distribution, and food security: an analysis for South Africa. Glob Food Sec. (2020) 26:100410. 10.1016/j.gfs.2020.100410 32834955 PMC7366977

[34] Jacobs M McDade TR Chaparro MV Corea M. Sick, hungry, and vulnerable: federal stimulus and food security on marginalized populations during the COVID-19 pandemic. J Racial Ethn Health Disparities. (2023) 10:2685–703. 10.1007/s40615-022-01447-8 36378487 PMC9666987

[35] Kwok S Adam S Ho JH Iqbal Z Turkington P Razvi S . Obesity: A Critical risk factor in the COVID-19 pandemic. Clin Obes. (2020) 10:e12403. 10.1111/cob.12403 32857454 PMC7460880

[36] Al Meslamani AZ Kassem AB El-Bassiouny NA Ibrahim OM. An emergency plan for management of COVID-19 patients in rural areas. Int J Clin Pract. (2021) 75:e14563. 10.1111/ijcp.14563 34165849 PMC8420210

[37] Morante-García W Zapata-Boluda RM García-González J Campuzano-Cuadrado P Calvillo C Alarcón-Rodríguez R. Influence of social determinants of health on COVID-19 infection in socially vulnerable groups. Int J Environ Res Public Health. (2022) 19:1294. 10.3390/ijerph19031294 35162317 PMC8834846

[38] Joseph J Sankar H Benny G Nambiar D. Who are the vulnerable, and how do we reach them? Perspectives of health system actors and community leaders in Kerala, India. BMC Public Health. (2023) 23:748. 10.1186/s12889-023-15632-9 37095483 PMC10123577

[39] Liu C Zhao Y Okwan-Duodu D Basho R Cui X. COVID-19 in cancer patients: risk, clinical features, and management. Cancer Biol Med. (2020) 17:519–27. 10.20892/j.issn.2095-3941.2020.0289 32944387 PMC7476081

[40] Parise R Li YE Nadar RM Ramesh S Ren J Govindarajulu MY . Health influence of SARS-CoV-2 (COVID-19) on cancer: a review. Acta Biochim Biophys Sin. (2022) 54:1395–405. 10.3724/abbs.2022147 36269132 PMC9828497

[41] Bigdelou B Sepand MR Najafikhoshnoo S Negrete JAT Sharaf M Ho JQ . COVID-19 and preexisting comorbidities: risks, synergies, and clinical outcomes. Front Immunol. (2022) 13:890517. 10.3389/fimmu.2022.890517 35711466 PMC9196863

[42] Azevedo RB Botelho BG de Hollanda JVG Ferreira LVL Junqueira de Andrade LZ Oei SSML . Covid-19 and the cardiovascular system: a comprehensive review. J Hum Hypertens. (2021) 35:4–11. 10.1038/s41371-020-0387-4 32719447 PMC7384729

[43] Soumya RS Unni TG Raghu KG. Impact of COVID-19 on the cardiovascular system: a review of available reports. Cardiovasc Drugs Ther. (2021) 35:411–25. 10.1007/s10557-020-07073-y 32926272 PMC7487338

[44] Nomah DK Reyes-Urueña J Llibre JM Ambrosioni J Ganem FS Miró JM . HIV and SARS-CoV-2 Co-infection: epidemiological, clinical features, and future implications for clinical care and public health for people living with HIV (PLWH) and HIV most-at-risk groups. Curr HIV/AIDS Rep. (2022) 19:17–25. 10.1007/s11904-021-00596-5 35113346 PMC8810339

[45] Garcia PJ Cabrera DM Cárcamo PM Diaz MM. HIV and COVID-19 in Latin America and the Caribbean. Curr HIV/AIDS Rep. (2022) 19:37–45. 10.1007/s11904-021-00589-4 35092570 PMC8799981

[46] Lesko CR Bengtson AM. HIV and COVID-19: intersecting epidemics with many unknowns. Am J Epidemiol. (2021) 190:10–6. 10.1093/aje/kwaa158 32696057 PMC7454306

[47] Chen Y Klein SL Garibaldi BT Li H Wu C Osevala NM . Aging in COVID-19: vulnerability, immunity and intervention. Ageing Res Rev. (2021) 65:101205. 10.1016/j.arr.2020.101205 33137510 PMC7604159

[48] Bansod S Ahirwar AK Sakarde A Asia P Gopal N Alam S . COVID-19 and geriatric population: from pathophysiology to clinical perspectives. Horm Mol Biol Clin Investig. (2021) 42:87–98. 10.1515/hmbci-2020-0053 33544506

[49] Martínez-Payá M Carrillo I Guilabert M. Lessons learned from the COVID-19 pandemic in nursing homes: a systematic review. Int J Environ Res Public Health. (2022) 19:16919. 10.3390/ijerph192416919 36554806 PMC9779143

[50] Tamara A Tahapary DL. Obesity as a predictor for a poor prognosis of COVID-19: a systematic review. Diabetes Metab Syndr. (2020) 14:655–9. 10.1016/j.dsx.2020.05.020 32438328 PMC7217103

[51] Yu W Rohli KE Yang S Jia P. Impact of obesity on COVID-19 patients. J Diabetes Comp. (2021) 35:107817. 10.1016/j.jdiacomp.2020.107817 PMC7690270 33358523

[52] Bertolini F Witteveen AB Young S Cuijpers P Ayuso-Mateos JL Barbui C . Risk of SARS-CoV-2 infection, severe COVID-19 illness and COVID-19 mortality in people with pre-existing mental disorders: an umbrella review. BMC Psychiatry. (2023) 23:181. 10.1186/s12888-023-04641-y 36941591 PMC10026202

[53] Tenenbaum A Glasbauer D Wexler ID. Coronavirus and people with intellectual disabilities: a special perspective. Isr Med Assoc J. (2021) 23:5–6. 33443333

[54] Murphy L Markey K O' Donnell C Moloney M Doody O. The impact of the COVID-19 pandemic and its related restrictions on people with pre-existent mental health conditions: a scoping review. Arch Psychiatr Nurs. (2021) 35:375–94. 10.1016/j.apnu.2021.05.002 34176579 PMC9759111

[55] De Hert M Mazereel V Stroobants M De Picker L Van Assche K Detraux J. COVID-19-related mortality risk in people with severe mental illness: a systematic and critical review. Front Psychiatry. (2022) 12:798554. 10.3389/fpsyt.2021.798554 35095612 PMC8793909

[56] Chagas GCL Rangel AR Noronha LM da Silva GB Jr Meneses GC Martins AMC . COVID-19 e os rins: uma revisão narrativa. Rev Bras Saude Mater Infant. (2021) 21:373–81. 10.1590/1806-9304202100s200003

[57] Dashraath P Wong JLJ Lim MXK Lim LM Li S Biswas A . Coronavirus disease 2019 (COVID-19) pandemic and pregnancy. Am J Obstet Gynecol. (2020) 222:521–31. 10.1016/j.ajog.2020.03.021 32217113 PMC7270569

[58] Rocha R Atun R Massuda A Rache B Spinola P Nunes L . Effect of socioeconomic inequalities and vulnerabilities on health-system preparedness and response to COVID-19 in Brazil: a comprehensive analysis. Lancet Glob Health. (2021) 9:e782–92. 10.1016/S2214-109X(21)00081-4 33857500 PMC8041360

[59] dos Santos MPA Nery JS Goes EF Silva A da Santos ABS dos Batista LE . População negra e Covid-19: reflexões sobre racismo e saúde. Estudos Avançados. (2020) 34:225–43. 10.1590/s0103-4014.2020.3499.014

[60] Brakefield WS Olusanya OA White B Shaban-Nejad A. Social determinants and indicators of COVID-19 among marginalized communities: a scientific review and call to action for pandemic response and recovery. Disaster Med Public Health Prep. (2022) 17:e193. 10.1017/dmp.2022.104 35492024 PMC9237492

[61] Tan SY Foo CD Verma M Hanvoravongchai P Cheh PLJ Pholpark A . Mitigating the impacts of the COVID-19 pandemic on vulnerable populations: lessons for improving health and social equity. Soc Sci Med. (2023) 328:116007. 10.1016/j.socscimed.2023.116007 37279639 PMC10234832

