# Supplementary material for: Mapping vulnerability for increased COVID-19 susceptibility and worse outcomes: a scoping review
Source: Front Public Health. 2024 Oct 10;12:1432370. doi: 10.3389/fpubh.2024.1432370 (PMC11499102; doi:10.3389/fpubh.2024.1432370)
Supplement: Supplementary file 1 [file Table_1.DOCX]

Supplementary Material

Supplementary table 1. Research terms used to search for scientific articles on different databases.

| Database | Term combination |
| --- | --- |
| PubMed/MEDLINE | (("Coronavirus Infections"[Mesh] OR "Coronavirus Infections" OR "Coronavirus"[Mesh] OR "Coronavirus" OR "SARS-CoV" OR "COVID-19"[Mesh] OR "COVID-19" OR "SARS-CoV-2"[Mesh] OR "SARS-CoV-2" OR "SARSCoV2" OR "SARS2" OR "COVID19" OR "COVID-2019" OR "COVID 2019" OR "SARS COV 2" OR "2019-nCoV" OR "2019ncov" OR "nCoV 2019") AND ("Social Vulnerability"[Mesh] OR "Social Vulnerability" OR "Vulnerable Populations"[Mesh] OR "Vulnerable Populations" OR "Health Vulnerability" OR "vulnerable group" OR "vulnerable groups") AND ("Risk groups" OR "risk group" OR "risk population" OR "risk populations" OR "Population Groups"[Mesh] OR "Population Groups" OR "Population Group" OR "Social Group"[Mesh] OR "Social Group" OR "Social Groups" OR "Peer Group"[Mesh] OR "Peer Group") AND (journal article[Publication Type])) |
| Embase (Elsevier) | ('coronavirus infections'/mj OR 'coronavirus infections' OR 'coronavirus'/mj OR 'coronavirus' OR 'sars-cov'/mj OR 'sars-cov' OR 'covid-19'/mj OR 'covid-19' OR 'sars-cov-2'/mj OR 'sars-cov-2' OR 'sarscov2' OR 'sars2' OR 'covid19'/mj OR 'covid19' OR 'covid-2019'/mj OR 'covid-2019' OR 'covid 2019'/mj OR 'covid 2019' OR 'sars cov 2'/mj OR 'sars cov 2' OR '2019-ncov'/mj OR '2019-ncov' OR '2019ncov' OR 'ncov 2019'/mj OR 'ncov 2019') AND ('social vulnerability'/mj OR 'social vulnerability' OR 'vulnerable populations'/mj OR 'vulnerable populations' OR 'health vulnerability' OR 'vulnerable group' OR 'vulnerable groups') AND ('risk groups' OR 'risk group'/mj OR 'risk group' OR 'population groups'/mj OR 'population groups' OR 'population group'/mj OR 'population group' OR 'social group'/mj OR 'social group' OR 'social groups' OR 'peer group'/mj OR 'peer group') AND [article]/lim AND ([english]/lim OR [portuguese]/lim OR [spanish]/lim) AND [2020-2023]/py |
| Scopus (Elsevier) | (("Coronavirus Infections" OR "Coronavirus" OR "SARS-CoV" OR "COVID-19" OR "SARS-CoV-2" OR "SARSCoV2" OR "SARS2" OR "COVID19" OR "COVID-2019" OR "COVID 2019" OR "SARS COV 2" OR "2019-nCoV" OR "2019ncov" OR "nCoV 2019") AND ("Social Vulnerability" OR "Vulnerable Populations" OR "Health Vulnerability" OR "vulnerable group" OR "vulnerable groups") AND ("Risk groups" OR "risk group" OR "Population Groups" OR "Population Group" OR "Social Group" OR "Social Groups" OR "Peer Group")) |
| Web of Science (Clarivate Analytics) | (("Coronavirus Infections" OR "Coronavirus" OR "SARS-CoV" OR "COVID-19" OR "SARS-CoV-2" OR "SARSCoV2" OR "SARS2" OR "COVID19" OR "COVID-2019" OR "COVID 2019" OR "SARS COV 2" OR "2019-nCoV" OR "2019ncov" OR "nCoV 2019") AND ("Social Vulnerability" OR "Vulnerable Populations" OR "Health Vulnerability" OR "vulnerable group" OR "vulnerable groups") AND ("Risk groups" OR "risk group" OR "Population Groups" OR "Population Group" OR "Social Group" OR "Social Groups" OR "Peer Group"))  Note: topic field |
| Lilacs | (("Infecções por Coronavirus" OR "Infecciones por Coronavirus" OR "Coronavirus Infections" OR "Coronavirus" OR "SARS-CoV" OR "COVID-19" OR "SARS-CoV-2" OR "SARSCoV2" OR "SARS2" OR "COVID19" OR "COVID-2019" OR "COVID 2019" OR "SARS COV 2" OR "2019-nCoV" OR "2019ncov" OR "nCoV 2019") AND ("Vulnerabilidade Social" OR "Populações Vulneráveis" OR "Vulnerabilidade em Saúde" OR "grupo vulnerável" OR "grupos vulneráveis" OR "Vulnerabilidad Social" OR "Poblaciones Vulnerables" OR "Vulnerabilidad en Salud" OR "grupo vulnerable" OR "grupos vulnerables" OR "Social Vulnerability" OR "Vulnerable Populations" OR "Health Vulnerability" OR "vulnerable group" OR "vulnerable groups") AND ("Grupos de risco" OR "grupo de risco" OR "Grupos Populacionais" OR "Grupo Populacional" OR "Grupo Social" OR "Grupos Sociais" OR "Grupo Associado" OR "Grupos de riesgo" OR "grupo de riesgo" OR "Grupos de Población" OR "Grupo Poblacional" OR "Grupos Poblacionales" OR "Grupo Paritario" OR "Risk groups" OR "risk group" OR "Population Groups" OR "Population Group" OR "Social Group" OR "Social Groups" OR "Peer Group")) |
| Scielo | (("Infecções por Coronavirus" OR "Infecciones por Coronavirus" OR "Coronavirus Infections" OR "Coronavirus" OR "SARS-CoV" OR "COVID-19" OR "SARS-CoV-2" OR "SARSCoV2" OR "SARS2" OR "COVID19" OR "COVID-2019" OR "COVID 2019" OR "SARS COV 2" OR "2019-nCoV" OR "2019ncov" OR "nCoV 2019") AND ("Vulnerabilidade Social" OR "Populações Vulneráveis" OR "Vulnerabilidade em Saúde" OR "grupo vulnerável" OR "grupos vulneráveis" OR "Vulnerabilidad Social" OR "Poblaciones Vulnerables" OR "Vulnerabilidad en Salud" OR "grupo vulnerable" OR "grupos vulnerables" OR "Social Vulnerability" OR "Vulnerable Populations" OR "Health Vulnerability" OR "vulnerable group" OR "vulnerable groups") AND ("Grupos de risco" OR "grupo de risco" OR "Grupos Populacionais" OR "Grupo Populacional" OR "Grupo Social" OR "Grupos Sociais" OR "Grupo Associado" OR "Grupos de riesgo" OR "grupo de riesgo" OR "Grupos de Población" OR "Grupo Poblacional" OR "Grupos Poblacionales" OR "Grupo Paritario" OR "Risk groups" OR "risk group" OR "Population Groups" OR "Population Group" OR "Social Group" OR "Social Groups" OR "Peer Group")) |
